# Bias Analysis on Public X-Ray Image Datasets of Pneumonia and COVID-19 Patients

**DOI:** 10.1109/ACCESS.2021.3065456

**Published:** 2021-03-10

**Authors:** Omar Del Tejo Catalá, Ismael Salvador Igual, Francisco Javier Pérez-Benito, David Millán Escrivá, Vicent Ortiz Castelló, Rafael Llobet, Juan-Carlos Peréz-Cortés

**Affiliations:** Instituto Tecnológico de Informática (ITI), Universitat Politècnica de València 16774 46022 Valencia Spain; Department of Computer Systems and Computation (DSIC)Universitat Politècnica de València 16774 46022 Valencia Spain; Department of Computing Engineering (DISCA)Universitat Politècnica de València 16774 46022 Valencia Spain

**Keywords:** Deep learning, COVID-19, convolutional neural networks, chest X-ray, bias, segmentation, saliency map

## Abstract

Chest X-ray images are useful for early COVID-19 diagnosis with the advantage that X-ray devices are already available in health centers and images are obtained immediately. Some datasets containing X-ray images with cases (pneumonia or COVID-19) and controls have been made available to develop machine-learning-based methods to aid in diagnosing the disease. However, these datasets are mainly composed of different sources coming from pre-COVID-19 datasets and COVID-19 datasets. Particularly, we have detected a significant bias in some of the released datasets used to train and test diagnostic systems, which might imply that the results published are optimistic and may overestimate the actual predictive capacity of the techniques proposed. In this article, we analyze the existing bias in some commonly used datasets and propose a series of preliminary steps to carry out before the classic machine learning pipeline in order to detect possible biases, to avoid them if possible and to report results that are more representative of the actual predictive power of the methods under analysis.

## I. Introduction

Chest X-ray (CXR) radiography is the most widely accepted imaging modality for detecting pneumonia and it is becoming crucial for tracking the clinical evolution of COVID-19 patients [1]. The COVID-19 disease is caused by Severe Acute Respiratory Syndrome Coronavirus 2 (SARS-CoV-2) and has become a global pandemic in a few months. Early diagnosis is a key factor due to the stealthy contagious nature of the virus and a lack of vaccines or effective treatments and, thus, it helps to prevent further spreading and to control it under the existing healthcare facilities. The small size of the acquisition devices, their ease of operation and their low cost make them more widely available than the Computer Tomography (CT) equipment, despite image quality and the diagnostic performance of CT are superior.

As a response to the COVID-19 outbreak, the scientific community has rapidly reacted and a lot of works using CXR images for COVID-19 detection have been published. The majority of them make use of well-known CNN architectures such as VGG [2], ResNet [3]–[5], SqueezeNet [3], [6], DenseNet [7] and also combine them with decision trees [8] and Support Vector Machines (SVM) [9]. Given the difficulty of obtaining COVID-19 samples, GAN networks have been used [10], [11] in order to enhance the performance. Moreover, other approaches [12], [13] based on multi-resolution methods report results that are comparable to those obtained by CNNs.

Machine learning models need large amounts of data which, in this case, are difficult to acquire, being the existing collections a mix of already well-known datasets and new COVID-19 image datasets. This heterogeneous mixture of observations provides more variety and usually reduces epistemic uncertainty. However, if these datasets, for instance, are not equally balanced (label-wise), they may induce a certain amount of dataset bias to the training phase. This happens when the images can be easily discriminated by features not relevant to the task, i.e. if the dataset inadvertently contains some distinctive features which are not related to the disease and are not shared among the source datasets. For instance, let’s assume an extreme case. Two image datasets are formed by two different classes, that is, dataset A made of class A samples and dataset B of class B samples. Let’s assume in most dataset A samples there is a white rectangle on the top right corner, and the true class features are not as trivial. Classifiers will focus on the easiest feature to discriminate between classes and not the true class features. Therefore, this leads to poor generalization; given a new dataset C full of class A, samples with no white rectangle will be misclassified.

We have detected significant biases in some of the most commonly used datasets intended for pneumonia and COVID-19 detection and we suspect that the accuracy reported in some studies might be due in part to them, and thus not directly related to the image features that could characterize the disease. These biases could arise, for example, when using some specific devices to acquire images of patients with a low probability of suffering the disease (mainly controls), and different ones for those patients with a high probability of suffering it (mainly cases). This could happen, for example, when most of the patients are screened in certain health services and highly suspicious patients are derived to a different area or, even worse, when, aiming to increase the number of controls or cases, a dataset is expanded with samples coming from significantly different origins and labeled with unbalanced class identifiers. In these cases, a CNN trained to discriminate between cases and controls could learn to differentiate images from different origins rather than finding features actually related to the disease.

Therefore, to effectively assess the performance of the classifier, there must exist a previous study of the dataset bias, so that the results can be validated. Thus, we present several studies to assess the validity of the results. The following datasets will be used to perform the experiments: BIMCV Padchest, CheXpert, RSNA and a COVID-19 image data collection that we will refer to as COVIDcxr, which will be further described in Section II-A.

The main contributions of this work are:

• To propose a bias analysis methodology to assert the validity of the results achieved on a dataset.
• To study the possible existence of bias in three broadly used pneumonia classification datasets.
• To study the effect of mixing several datasets.

**FIGURE 1.**
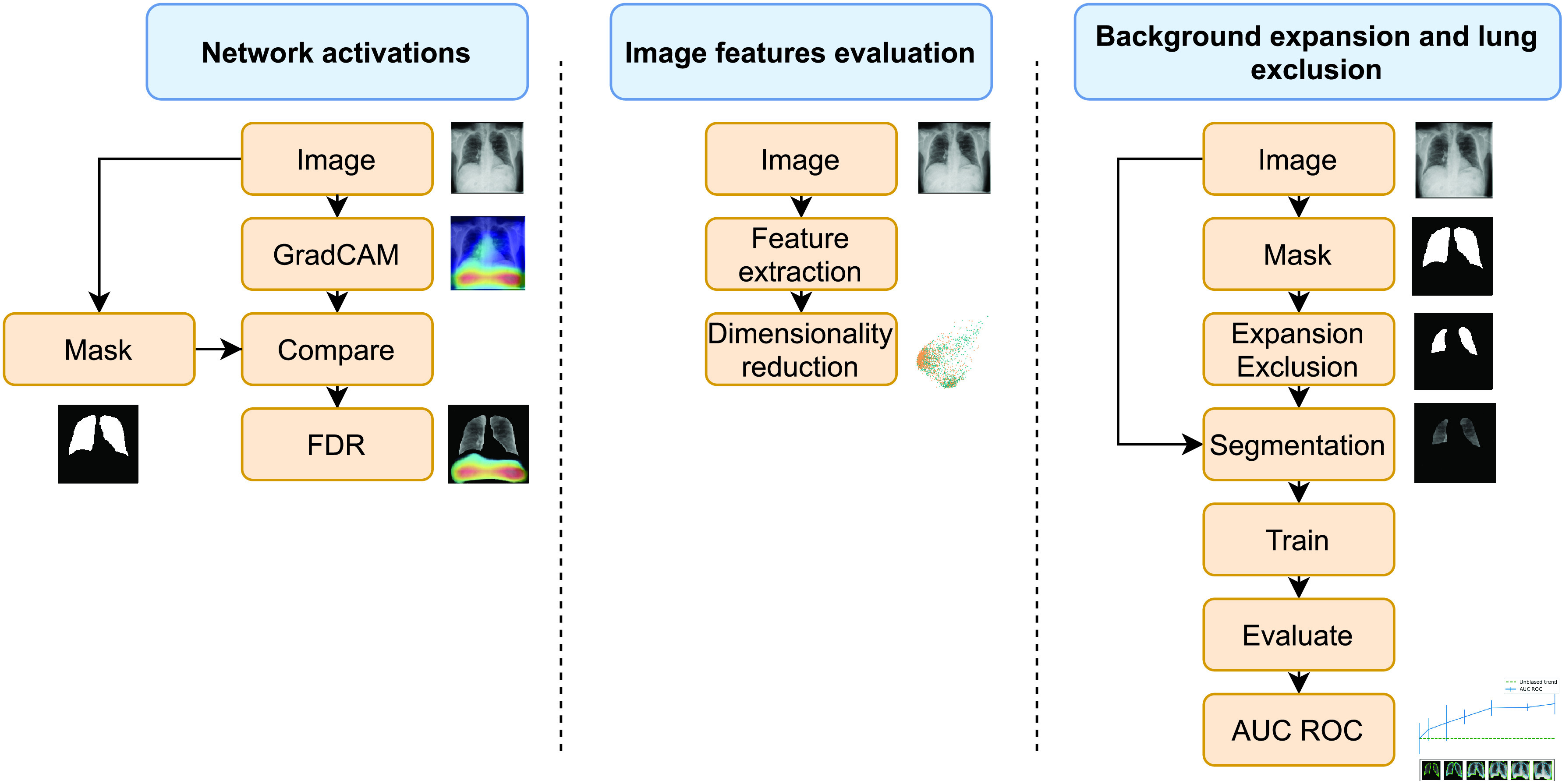
Workflow of the different experiments. From left to right: Network activations, Image features evaluation and background expansion and lung exclusion.

This work is structured as follows: Section I outlines the problem of bias in CXR datasets. After that, the datasets and networks used, along with the proposed methodology are described in Section II. The workflow related to this section can be seen in Figure 1. Section III shows the results achieved using this article’s methodology over the proposed datasets and Section IV gives an analysis of the results. Finally, conclusions are presented in Section VI.

## II. Methods

### A. Datasets

Several public datasets have been used in this article:

• PADCHEST^1^ [14] is a CXR dataset that includes more than 160K images from 67625 patients that were reported by radiologists at Hospital de San Juan (Spain) from 2009 to 2017. The reports are labeled with 174 different radiographic findings, 19 differential diagnoses and 104 anatomic locations. 27% of the reports were manually annotated by trained physicians and the remaining set was labeled using a supervised method based on a Recurrent Neural Network with attention mechanisms. Generated labels were validated, achieving a 0.93 Micro-F1 score using an independent test set. For the experiments, only Posterior-Anterior images are considered. Therefore, there are 9110 images in the remaining dataset: 6790 control and 2320 pneumonia images.
• RSNA pneumonia dataset^2^ is made up of images from the National Institutes of Health (NIH) and labeled by the Radiological Society of North America along with the Society for Thoracic Radiology and MD.ai. The goal of this dataset was to develop an AI classifier capable of distinguishing between pneumonia and control images, so it was released in a Kaggle competition in 2018. It consists of 26684 images from which 20672 are control and 6012 are pneumonia images.
• CheXpert dataset^3^ [15] is provided by Stanford University and contains 224316 chest radiographs of 65240 patients with labels of 14 sub-categories. The exams were performed at Stanford Hospital between October 2002 and July 2017. Structured labels for the images were created by an automated rule-based labeler, which the researchers developed to extract observations from free-text radiology reports. From the 224316 chest radiographs, this article only takes the ones related to pneumonia and control cases. Therefore, 5870 images are remaining in the dataset: 4878 control and 992 pneumonia images.
• COVID-19 image data collection (COVIDcxr)^4^ [16] is a project to collect X-ray and CT images that present COVID-19, SARS, MERS and ARDS from online sources. These sources are varied: scientific publications, websites, etc. As of June 2020, COVIDcxr has around 424 COVID-19 images and is one of the largest COVID-19 datasets publicly available to the best of our knowledge.

^1^http://bimcv.cipf.es/bimcv-projects/padchest/^2^https://www.kaggle.com/c/rsna-pneumonia-detection-challenge^3^https://www.healthimaging.com/topics/artificial-intelligence/stanford-researchers-release-chest-x-ray-dataset-train-ai^4^https://github.com/ieee8023/covid-chestxray-dataset

### B. Motivation

**FIGURE 2.**
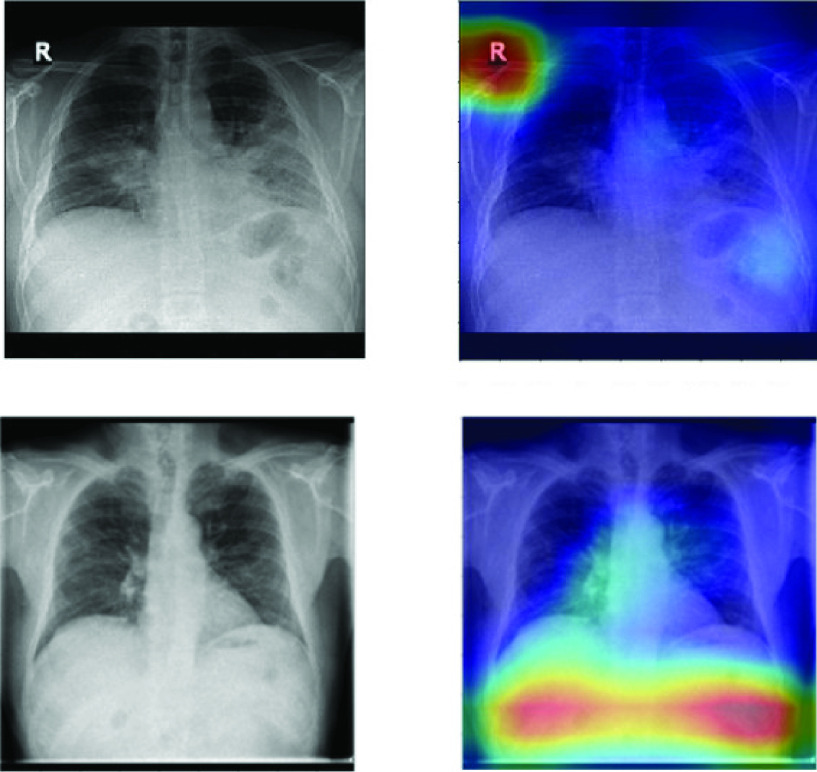
Lung heatmaps for BIMCV’s dataset.

The motivation for this study comes from analyzing the results of a neural network trained to classify between radiographic images of patients with pneumonia and healthy control patients in order to determine the validity of the classification. An interesting first validation can be done by visualizing the network’s activation heatmaps. When we performed these checks against networks trained with pneumonia datasets, we observed many suspicious patterns, as these heatmaps often highlighted areas of the image which did not contain lung tissue (see Figure 2). This made us suspect that the networks were learning to classify, achieving large values of AUC ROC, using features unrelated to the task. Thus, the datasets might be biased.

Grad-CAM [17] allows us to visualize the gradient of the label in the final convolutional layer to produce a heatmap depicting regions of the image that are relevant for the prediction. Blue pixels and red pixels correspond to low and high values of the gradient at the final convolutional layer, respectively.

As observed in Figure 2, there are highly activated regions in areas without lung presence when the expected activation should be inside the lung. It is not known how many pixels inside the lungs should show an activation, as no detection mask is available. However, we can assume that the activation map in a control patient should not exceed a given threshold, whilst a positive case’s map should show widespread activations within the lungs. Nonetheless, the activated area outside the lungs should be minimal in all cases. For this reason, a measure to inform about the distribution of the activated pixels could be useful.

Given a heatmap image $I=\{p_{ij}\}\in \mathrm {Mat}_{n,m}(\mathbb {R})$, where ${n}$ is the number of rows, ${m}$ the number of columns, and $p_{ij}$ represents the pixel value at row ${i}$ and column ${j}$. Let ${A}$ be a region of interest and ${B}$ its complement. Let ${t}$ be the activation map threshold, and let ${R}$ and ${W}$ be the number of pixels with an activation value higher than ${t}$ that are in ${A}$ and ${B}$ respectively.

**FIGURE 3.**
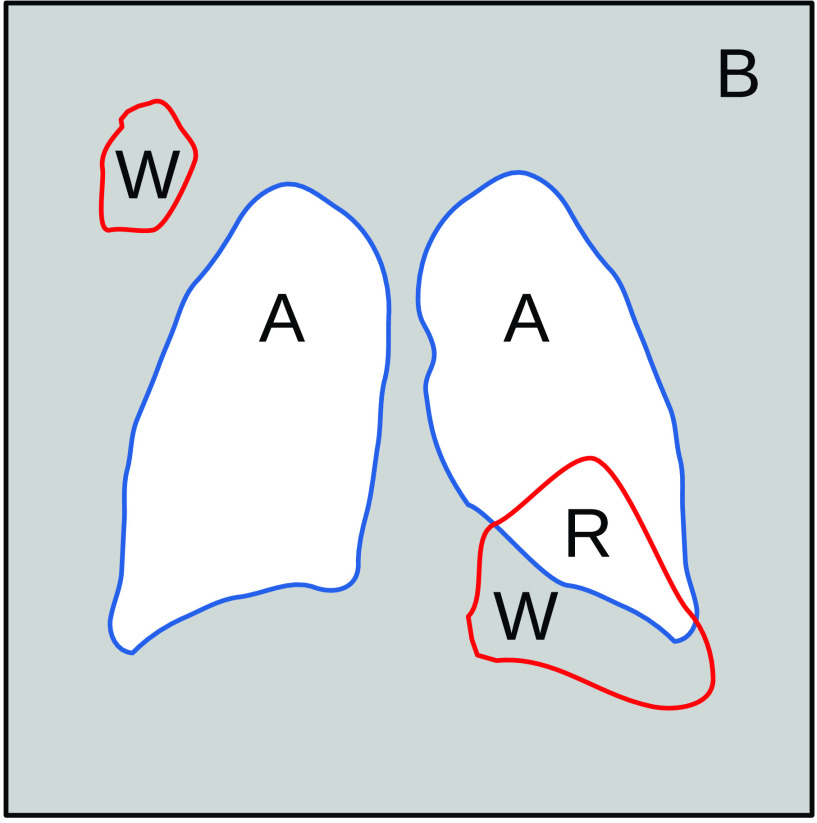
Activation regions diagram.

We can calculate the percentage of pixels with an activation value over a threshold that fall outside an expected region as the quotient between $W$ and $W+R$ (see Figure 3 and the equations below, where $p \in \{p_{ij}\}=I$).

Considering activated pixels in region ${W}$ as false positives (FP) and activated pixels in region ${R}$ as true positives (TP), the above quotient corresponds to the False Discovery Rate (FDR), which is the complement of the Positive Predictive Value (PPV).

\begin{align*} R=&\mathrm {TP}=|(p > t) \cap A| \\ W=&\mathrm {FP}=|(p > t) \cap B| \\ \mathrm {FDR}=&\frac {\mathrm {FP}}{\mathrm {FP}+\mathrm {TP}} \\ \mathrm {FDR}=&1-\mathrm {PPV}\end{align*}

For instance, in this task, any activated pixel that falls outside the lungs is marked as wrong ( ${W}$), as no information should be found there. The lower this value, the better. This score is designed to measure the validity of the trained CNN classifier based on its activation maps and allows the selection of different operation points depending on the threshold ${t}$ to be applied to the heatmaps. In this work, $t$ is set to 90% of the maximum heatmap value.

Table I shows the computed FDR for the activation maps under three different datasets. It is worth noting that some image findings are usually located on the border of the lungs, so if the highlighted area is near the border, some pixels might easily fall outside the region ( ${A}$) and be considered as wrong ( ${W}$). On the grounds of the information provided by the FDR, further experiments would be required to measure the extent to which this phenomenon affects the datasets.

**TABLE 1:** False Discovery Rate of Activation Maps for Three Different Datasets

	FDR
BIMCV	71.83%
RSNA	40.46%
CheXpert	50.68%

Additionally, some suspicious patterns appeared when visualizing the grayscale histograms of the images. Ideally, gray levels of images from different sources should be equally distributed, but in practice, this may not happen and give rise to inaccurate conclusions. The histograms of the images may be considered as Probability Density Functions (PDFs) and may serve to measure the variability among gray-level distributions using a methodology based on information geometry [18]. This methodology has been successfully applied to characterize EHR (Electronic Health Record) data [19], [20], to assess the variability among patients with different headache pain intensity [21], or to detect pixel distribution differences among images acquired from different mammographs [22].

Given a set of PDFs, this approach is based on the computation of the distance between each pair of PDFs using the Jensen-Shannon distance. The simplex where each point represents a PDF and the distance between two points is the Jensen-Shannon distance between the two PDFs they represent is known as a statistical manifold, which in turn is a Riemannian manifold. For visualization purposes, this simplex may be embedded in a real Euclidean space by using Multidimensional Scaling [23] and, finally, projected into two dimensions using a dimension reduction algorithm such as Principal Component Analysis.

**FIGURE 4.**
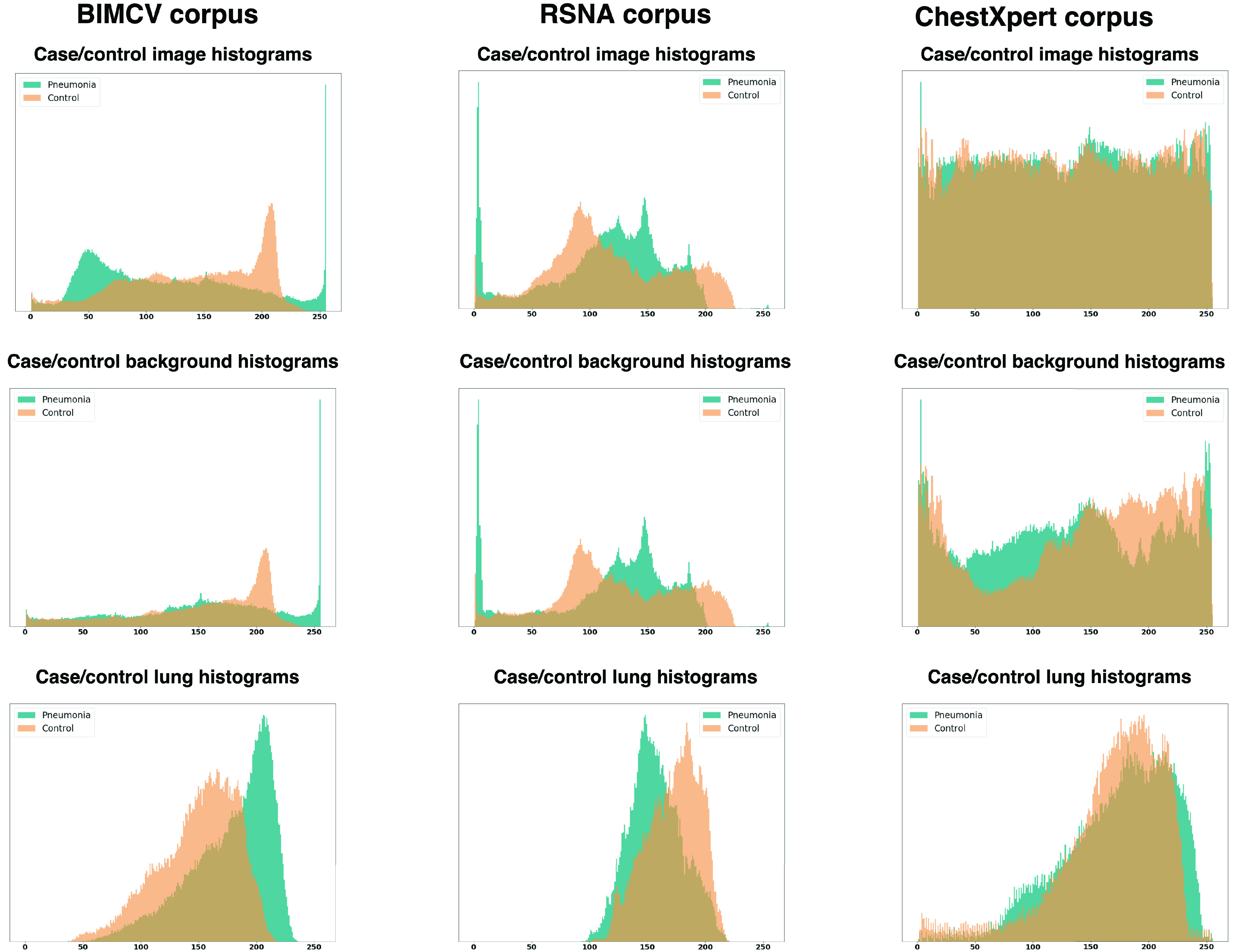
Example of case and control patient histograms. The first row shows the histogram of the whole image for an example of a case and a control patient, the second row shows the histogram of the background (the image with the lung area subtracted) and, the last one shows the histogram of the lungs.

**FIGURE 5.**
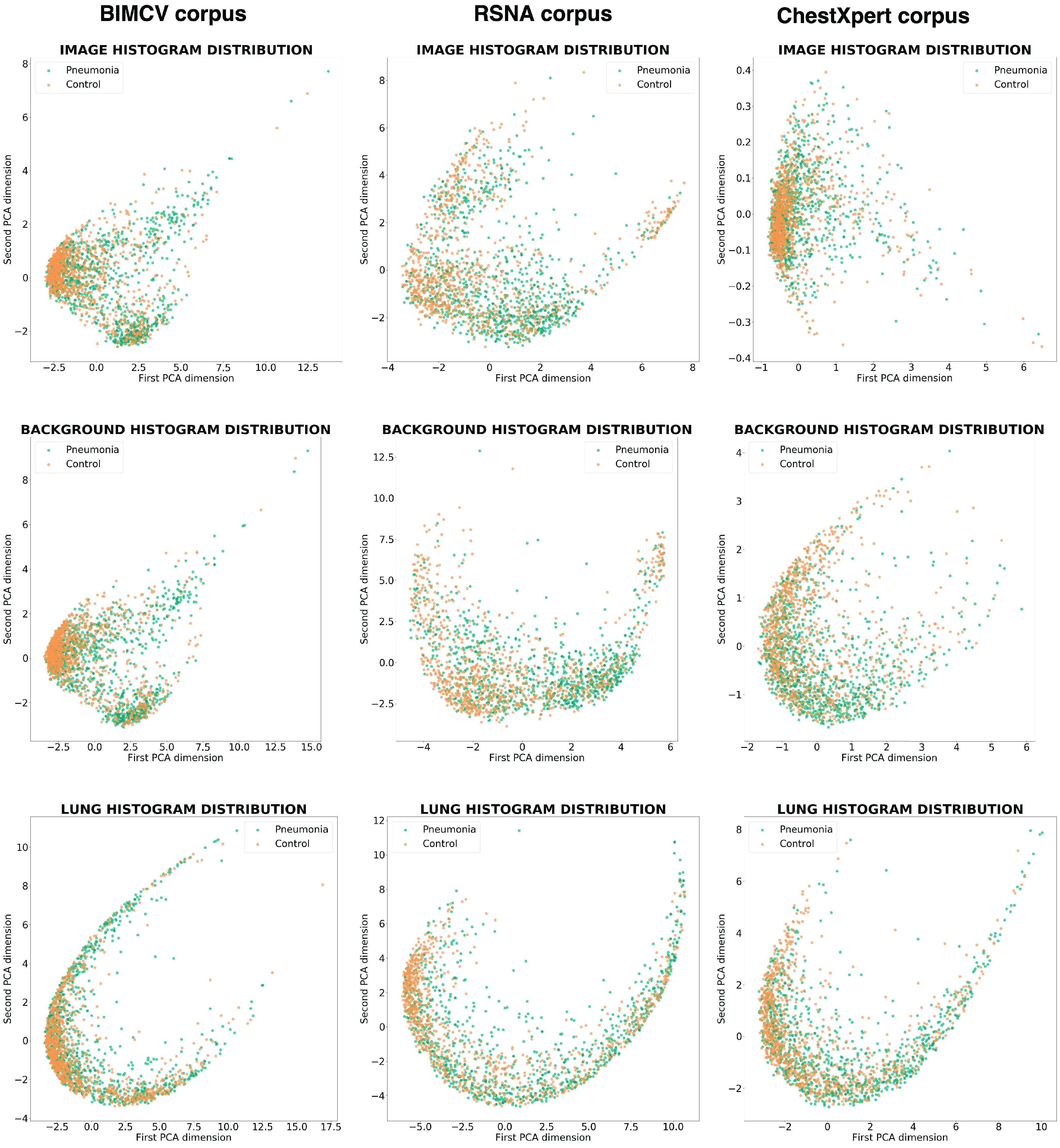
Image histogram variability. The first row represents the variability of the histograms of the complete images, the second row the variability of the background histograms (the images with the lung area subtracted), and the third row the histograms of the lungs. The first column represents a sample of BIMCV’s dataset, the second column a sample of RSNA’s, and the last, a sample of CheXpert’s.

This methodology was applied three times to a random balanced sample of 2000 individuals (1000 pneumonia cases and 1000 controls) of each dataset mentioned, which will be described in section II-A. Firstly, it was applied to the histograms of the complete images and, after a segmentation step, which will be described in detail in section II-D, the variability analysis was applied only to the histograms of the backgrounds, and then to the histograms of the lungs (see Figure 4). The variability of the three datasets is shown in Figure 5.

In the center row of Figure 5, which depicts the distributions of the backgrounds of the different datasets, we can see that the first two columns show distinct clusters composed predominantly of cases or controls that allow a certain degree of discrimination without taking into account the lung tissue. In fact, the last row, which represents lung area, shows fewer differences between the cases and control patient histograms. In the last column, corresponding to CheXpert’s dataset, these differences are not evident.

This could imply that, for some datasets, as BIMCV and RSNA, a Machine Learning algorithm can classify pneumonia and control cases using features outside the lungs.

### C. Network

In this article, Convolutional Neural Networks (CNNs) are used to classify the CXR images. These Machine Learning models have been widely employed in the last years for image classification, particularly in the field of medical imaging. The CNN topology used is VGG16 [24], which is broadly reported as a good classifier for chest image analysis [25]–[27]. In this scenario, a common practice with this type of networks is to trim the last layers (usually dense layers) and add a lighter classifier, which in this case is a Global Average Pooling followed by a Multilayer Perceptron, which projects the pooled features of VGG’s last convolution to 64 dimensions before performing the classification.

Transfer learning technique is a common practice within Deep Learning models. It is proven that pretrained networks, in particular their first layers, are generic and can be transferred to new domains without requiring special training. In fact, it also facilitates training for domains with a scarce amount of training samples. Therefore, the VGG16 network used is pretrained with Imagenet dataset, and the last 2 convolutional layers, along with the classification layers, are unfrozen for domain training.

It is noteworthy that the network structure is, up to a point, not critical for the conclusions drawn in this article, as it is not trying to present advancement in the state-of-the-art classification for the datasets used. The focus is rather on comparing the results obtained for images coming from different datasets, and whether those results suggest the presence of classification biases within the data. Nonetheless, it must at least achieve an acceptable accuracy in order to ensure the extracted features are good enough and close to the ones extracted in other articles.

### D. Segmentation

By segmenting the lungs, it is possible to remove parts of the image that do not contain relevant information and that can be a source of noise or bias, such as the presence of text annotations that can identify a machine or a hospital, or the appearance of images coming from specific medical devices that have been used in more cases than control patients or vice versa.

**FIGURE 6.**
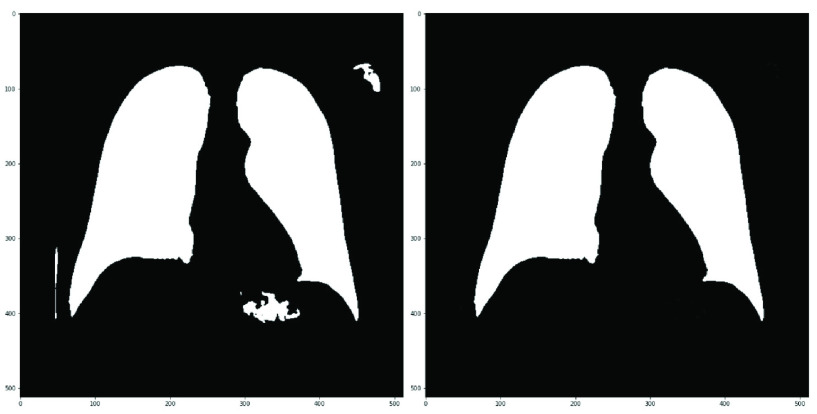
Lung segmentation (left) and after post-process (right).

Lung segmentation in CXR images has been successfully tackled with different approaches during the last years [28]. For this work, a U-Net network has been trained on the Montgomery dataset [29]. Moreover, we have manually labeled a total of 1115 images coming from BIMCV’s Padchest dataset to increase the number of training images. Figure 6 shows the segmentation results. This network achieves 0.974 DICE and 0.934 IoU scores over the Montgomery test partition, where DICE and IoU are defined as follows, being $A$ and $B$ the predicted segmentation mask and the true segmentation mask.

\begin{align*} \mathrm {DICE}=&\frac {2\mid \mathit {A}\cap \mathit {B}\mid }{\mid \mathit {A}\mid +\mid \mathit {B}\mid } \\[-3pt] \mathrm {IoU}=&\frac {\mathit {A}\cap \mathit {B}}{\mathit {A}\cup \mathit {B}}\end{align*}

### E. Bias Analysis

This work proposes a methodology to measure the degree of bias in a dataset. The focus is on the classification of pneumonia or COVID against control samples, but the methods can be generalized to other classification tasks where prior knowledge of the region of interest is available.

As stated before, areas that should not contain information about the problem can be possibly used to discriminate between classes, for example, text annotations or image features related to the medical devices employed. In order to solve this problem, we make use of a segmentation algorithm to extract the relevant regions which in this case are the lungs (see Figure 6). These regions will be referred to as masks. The rest of the image will be considered as background (see ${B}$ in Figure 3).

**FIGURE 7.**
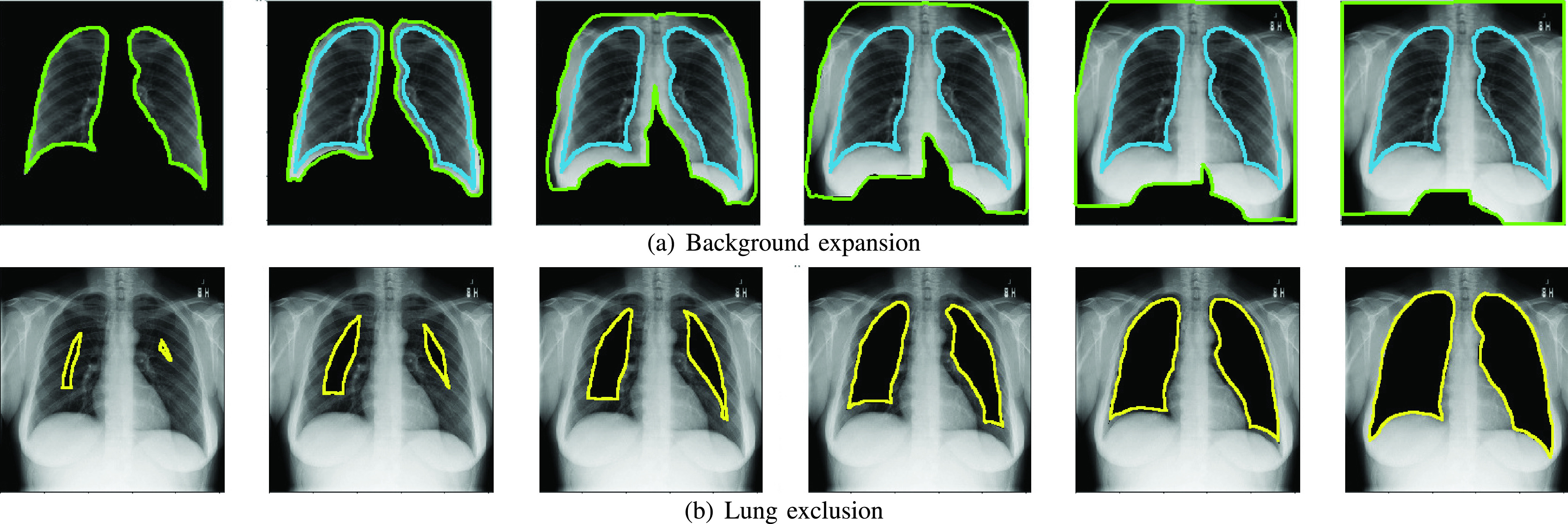
Background expansion and lung exclusion. (a) The original contour area is shown in blue and the expanded area contour in green (b) The contour of the removed area is shown in yellow.

To check the previous hypothesis presented in II-B, two experiments were carried out by training a model with different image areas according to the following ideas:

• We want to study how the background affects the results. Starting from an image that contains only the lungs (the background is erased), the visible region is progressively expanded to include more background by means of sequential dilation operations over the mask (see Figure 7a). An unbiased dataset should not increase the classification accuracy along this process.
• We want to analyze how the lack of lung area affects the results; this time starting from the whole image and progressively removing the lungs (see Figure 7b). The classification accuracy over an unbiased dataset should progressively drop from its maximum value (whole image) to 0.5 AUC ROC.

Thus, adjusting the expansion or exclusion of the lung region will allow us to trace the variation of the accuracy metric. We used images scaled to $256\times 256$ pixels. For background expansion, lung segmentation masks were dilated 0, 10, 30, 50, 80, 120 and 140 pixels and for lung exclusion, masks were eroded 0, 10, 20, 30, 40 and 100 pixels (from right to left in Figure 7).

**FIGURE 8.**
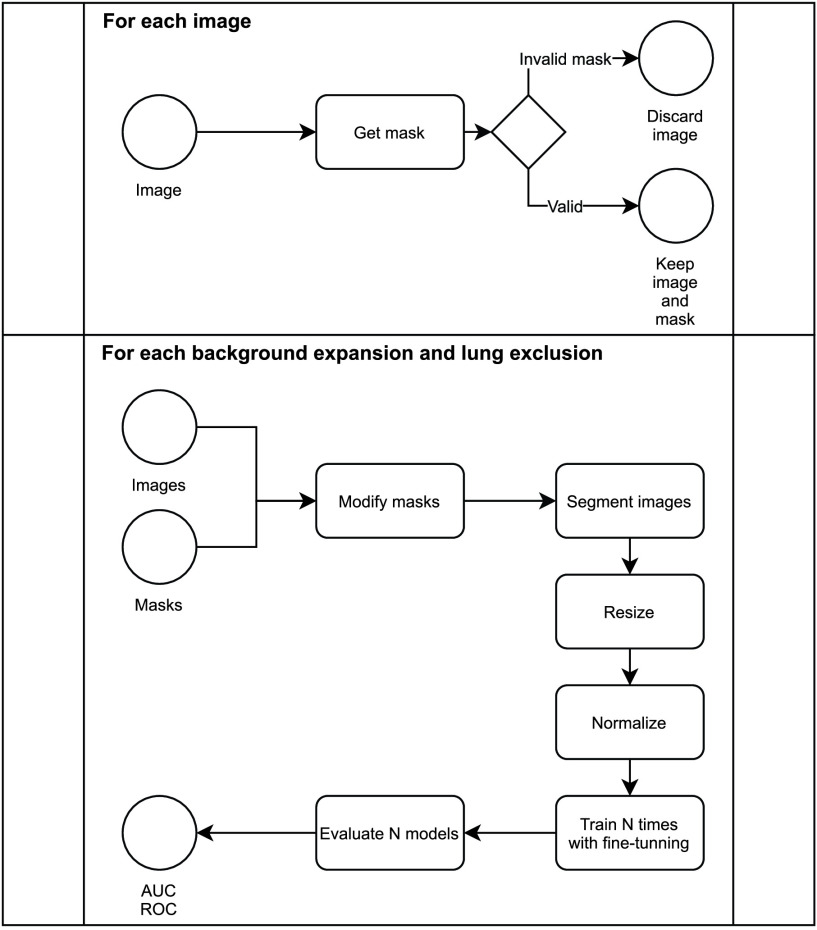
Bias analysis’s workflow.

Figure 7a shows the lung segmented area in blue and the background expansion in green. Also, Figure 7b shows the lung exclusion area in yellow. Additionally, a detailed workflow for this experiment is shown in Figure 8

### F. Combination Analysis

Combining datasets can be useful to enlarge the sample size, increase the variability explained by the data, and reduce the epistemic uncertainty of the classifiers. This latter is related to the problem-domain knowledge of the model, being it the uncertainty or lack of knowledge bound to the limited amount of data. However, if the combination and the balance among the classes are not carefully controlled, a classifier may learn to discriminate between features of the different datasets.

To check this hypothesis, we mixed RSNA and CheXpert datasets to achieve a balanced combination by adding positive pneumonia observations from the RSNA dataset into CheXpert. The latter is a highly unbalanced dataset (83% of negative and 27% of positive observations after our pre-process and segmentation validity filters), so it could be considered a good idea to add positive samples from another dataset. Needless to say, if the images from RSNA have distinct features that allow the classifier to tell them apart from CheXpert, for example including a large proportion of images from a particular equipment brand or model, the system will learn to classify the images from that equipment as positive, regardless of any image content that could be related to the disease.

Additionally, we simulated the combination of COVID-19 and control datasets and evaluated their bias with the proposed method. In particular, the datasets combined are positive COVID-19 cases from COVIDcxr with CheXpert’s negative control samples. COVIDcxr is built with datasets from different origins, hence this experiment illustrates the likely problematic effects of heterogeneous data combinations.

Based on our methodology that probes the discrimination induced outside the lungs, the expectations about the results of the experiment, if there is bias in the dataset, are: (1) the background expansion could increase the accuracy and (2) the accuracy when occluding the lungs should differ significantly from the 0.5 AUC ROC. Did the results follow these predictions, the hypothesis would be confirmed.

## III. Results

### A. Background Expansion and Lung Exclusion Study

In the previous section, we proposed to examine the performance of classification experiments varying the addition of background and the reduction of the lung area. The expected results of the first test for a non-biased dataset, where the background area is added to the initial lung-only images, is that the classification rate stays constant (or almost constant, due to possible imprecise segmentation and other random perturbations), as the disease information is already present from the beginning.

**FIGURE 9.**
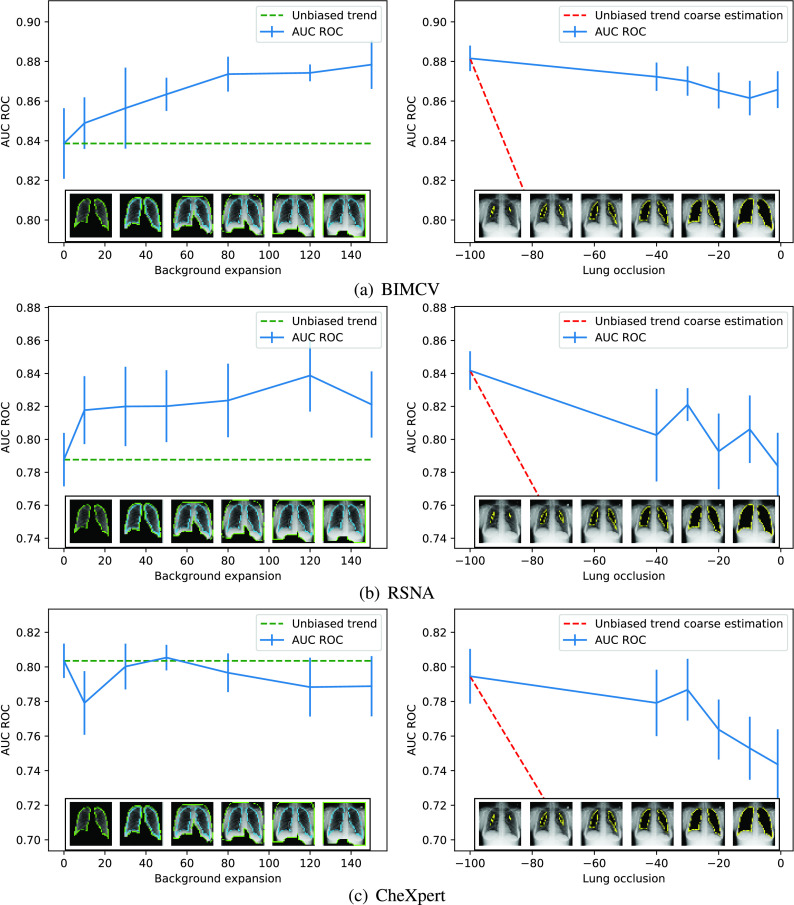
Accuracy as a function of the background expansion and lung reduction. The green dotted lines mark the correct behavior of a non-biased dataset when more and more background is included, and the red dotted lines indicate the expected reduction of the classification rate as the lungs are removed from the analysis. Blue lines show the accuracy for a given expansion or reduction with a vertical line indicating the standard deviation.

In the second scenario, the accuracy should potentially drop from the value achieved when the network sees the complete image to a value close to 0.5 AUC ROC when the lungs are completely removed. This drop is not necessarily linear, but will be shown in the graphs as a straight red line, as can be seen in the right part of Figure 9, to offer a simplified graphical representation of the expected behavior. In the left part of the figure, the green line represents the classification rate obtained using only the lung area.

This analysis has been performed in the three datasets:

• The first one (see Figure 9a), BIMCV, clearly shows a significant bias within the data, as the classification rate steadily increases with the background expansion. The second graph shows that removing the lung area is not associated with a significant decrease in accuracy, as it should, and even with the complete exclusion of the lungs the classifier achieves almost 0.88 AUC ROC.
• The second one (see Figure 9b), RSNA, displays a slightly lower but still consistent bias within the data in both graphs. However, the RSNA dataset was harder to segment than the other ones and, thus, part of the variability shown could arise from poorly segmented images. Nonetheless, a 0.79 AUC ROC is achieved with the lungs completely occluded, which is far from the expected 0.5 AUC ROC.
• The third one (see Figure 9c), CheXpert, conveys interesting results. The left graph’s trend is the one expected for an unbiased dataset, as it doesn’t vary along with the background expansion. Nevertheless, the precision achieved when the lung is completely occluded is around 0.74 AUC ROC. This implies that the bias is not located specifically in the background, but it must lie in the whole image.

### B. Combination Study

As mentioned before, the combination study seeks to evaluate how the combination of datasets might provoke the creation of biased data and how the methodology proposed can detect these weaknesses in the final data collection.

**FIGURE 10.**
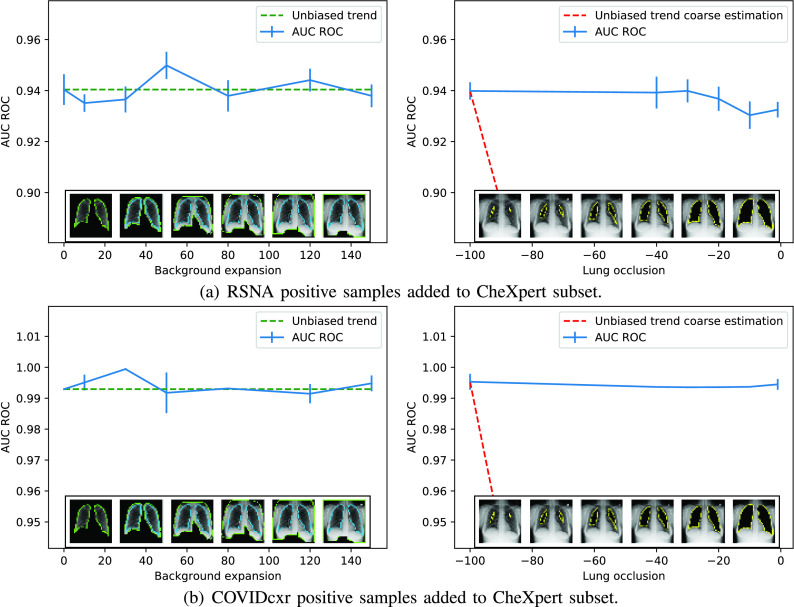
Addition of positive samples from RSNA and COVIDcxr to CheXpert’s dataset. The green dotted lines mark the correct behavior of a non-biased dataset when more and more background is included, and the red dotted lines indicate the expected reduction of the classification rate as the lungs are removed from the analysis. Blue lines show the accuracy for a given expansion or reduction with a vertical line indicating the standard deviation.

The experiments of Section III-A have been reproduced using the combined dataset. Figure 10(a) shows the effect of varying background expansion and lung exclusion when the combination is designed to balance CheXpert with RSNA cases (4878 control and 992 positive pneumonia images from CheXpert plus 3886 positive images from RSNA, giving a balanced dataset with 50% observations from each class).

The last experiment explored a combination of 4878 images of control patients from CheXpert and the whole set of 424 COVID-19 images from COVIDcxr. This dataset combination is typical of the recent crisis scenario, where few images from the new disease are available, they are obtained from different locations, under uncontrolled conditions, with different equipment and acquisition protocols, etc. This is the worst-case scenario and the results are in accordance with it, as can be seen in Figure 10(b).

The results for these experiments show, in a similar fashion to Chexpert’s base case, that the bias is ubiquitous in the image. Despite increasing the amount of background inside the images doesn’t affect the accuracy, the effect of the lung occlusion is not remarkable within the results.

## IV. Discussion

Deep learning has been receiving a lot of attention as a very powerful methodology for analyzing medical images [30]. The ability of Convolutional Neural Networks (CNN) to obtain excellent results even when it is used as a blackbox, as opposed to the classical design of ad-hoc algorithms, has attracted many researchers.

Some works using CNNs for COVID-19 detection on cxr images report high accuracies for a variety of network architectures. In particular, studies using VGG16 report [9] 89.8% accuracy for a dataset built of 180 COVID-19 and 200 control samples, 90% accuracy is obtained [27] for a dataset composed of 202 COVID-19 images, 300 of pneumonia and 300 negative and 93.48% accuracy [31] is achieved using a dataset that contains 224 COVID-19 images, 700 of pneumonia and 504 negative. The fact that VGG16 achieves good results for detecting pulmonary diseases strengthens the hypothesis that the features extracted by the network are relevant to the task and therefore, as detected from our experiments, related to some sort of bias within the images.

One of the drawbacks of CNNs is that they often need large amounts of data to learn and, while generic CXR databases are available, public existing COVID-19 datasets are composed of a few images that were collected by volunteers [16]. As a consequence, these datasets show unbalanced labels and a mix of different data sources that makes getting a robust model and reliable performance measures difficult. In this regard, some articles report the problem of small and unbalanced datasets for COVID-19 detection [4], [32], and propose solutions to mitigate the problem.

Bias analysis has been tackled by other authors. For instance, in [33] the authors proposed that train and test partitions should come from different datasets (related to the same task), as the classifier is trying to achieve maximum performance over a certain task and not over a dataset. This may also assert the true generalization capacity of the classifier. On the other hand, [34] sought to minimize the effects of different biased datasets by way of converting different dataset observations to prototypes, greatly reducing possible intra-dataset specific features.

Recently, [35] addresses this issue for COVID-19 detection and reports that the problem of mixing different datasets may lead the network to learn background information. Our study performs a similar approach to the one presented in thisarticle, i.e. both study possible biases within the lungs. [35] occludes the lungs with rectangular fixed-size black boxes and measures the accuracy achieved. However, the proposed methodology extends the concept proposed to more precise masks and progressive inclusion and exclusion of information to the learning process. This allows the ability to detect where the bias approximately is and enables more precise bias estimation.

Furthermore, [36] studies bias within the nCov2019 dataset using information about patients (symptoms, comorbidities, age, and sex). This dataset collects clinical data from different sources rather than images. They found significant bias related to the origin of the data and exposed several issues related to multisource variability.

This article is focused on detecting some biases within widely used CXR datasets to glimpse the degree to which these biases affect the results and proposes a bias detection methodology to assert the validity of results. This methodology makes use of techniques such as heatmap visualization, histogram analysis and selective image occlusion which are combined to evaluate which parts of the images are being used as discriminative features for a classification task. In this work, this methodology has been applied in two case scenarios, one for the existence of bias on individual pneumonia datasets and another to detect the existence of bias in a mix of datasets.

## V. Limitations of the Study

Regarding possible limitations, there could be a problem with the methodology proposed, since the segmentation masks used for expansion and reduction may be biased themselves. The segmentation process might be more prone to fail in images with pneumonia since the borders of the lungs are more diffuse, whereas this could not happen in images of control patients. This could pose a significant difference between cases and controls masks, and therefore, we might be introducing a new bias that would imply a problem with the proposed methodology.

**FIGURE 11.**
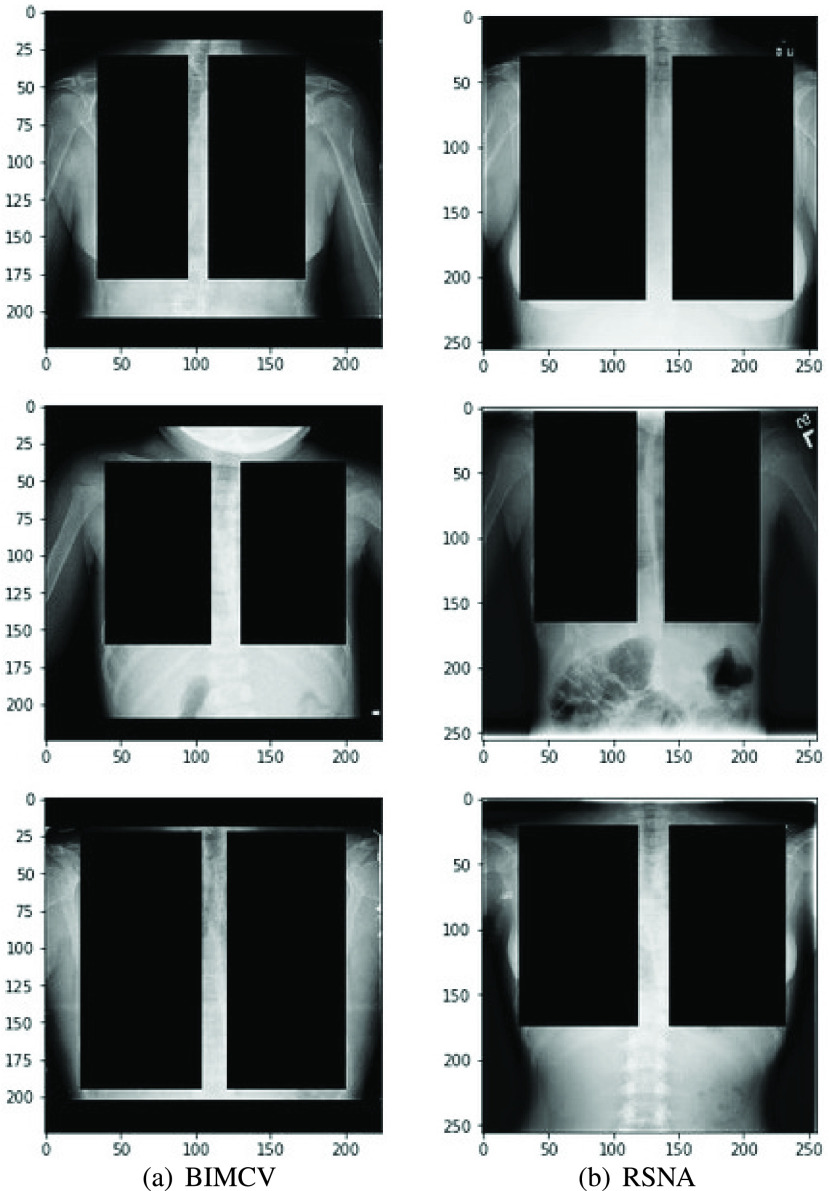
Lung occlusion with fixed-size rectangular boxes.

**FIGURE 12.**
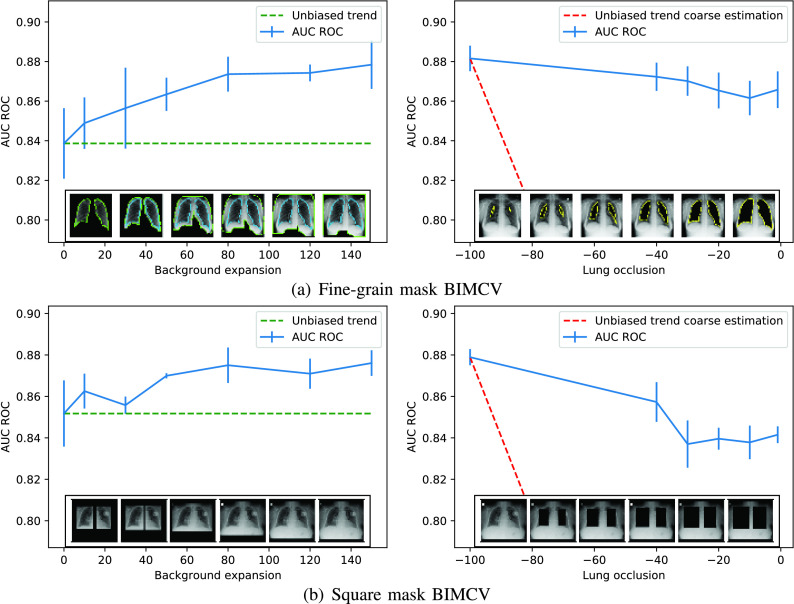
Comparison between fine-grain and squared masks for BIMCV’s dataset.

However, to rule this out, we designed an experiment where the occlusion masks were substituted by rectangles the size of the lungs. This experiment is similar to the one presented in [35], but here we ensure that the lungs are completely removed using the segmentation mask shape whereas in the aforementioned work they just place a fixed size black rectangle in the central area leaving some lung area uncovered. Some examples from our method can be seen in Figure 11. The results achieved for BIMCV’s dataset can be seen in Figure 12, where the differences found are not significant, suggesting that the shape of the lung masks is not influencing the bias detection algorithm proposed.

**FIGURE 13.**
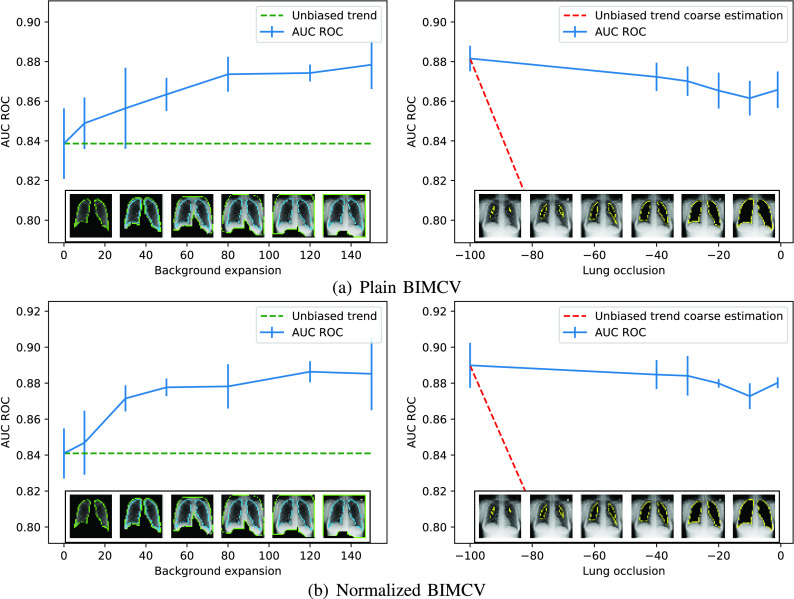
Comparison between normalized and plain BIMCV’s dataset.

Furthermore, to increase the confidence in our conclusions, we pre-processed all the images by means of CLAHE histogram normalization to assert how this pre-process affected the results. As can be seen in Figure 13, there is no difference in the results achieved between the normalized and plain images.

Talking about strengths, the results of the experiments described in Section III-B demonstrated that the classification rate does not improve when the background area is included in the images, which means that either there is no bias specifically on the background or the most significant bias is already within the lungs. However, when the lung area is progressively removed from the image we find in both experiments that the accuracy does not decrease, suggesting that the system is classifying the images according to some elements present in the whole image, not only inside the lungs. That result confirms the hypothesis that powerful systems like Convolutional Networks can find subtle features in the images and give optimistic classification results if no measures are taken to avoid biases in the data.

To summarize, further research should be conducted to reduce the impact of the intrinsic bias for the datasets whose images are collected from several sources. Recent literature has demonstrated the emergence of methodologies useful to reduce the impact of such a bias. Image preprocessing methods [22] or deep learning architectures designed to deal with biased datasets [37] may be a good starting point.

## VI. Conclusion

In this work, a novel methodology to assess the existence of bias in CXR image datasets is presented. Techniques such as activation heatmap visualization, histogram analysis and selective image occlusion are combined to evaluate which part of the images are being used as discriminative features for a classification task. In this case, the regions of interest were the lungs. The datasets used show different levels of bias, these comprising datasets that try to make information quickly available in an urgent scenario like the current COVID-19 crisis. Some examples are BIMCV’s collection or the combination of datasets created for this purpose, which are the ones with more problems. The results are confirmed with the other methodologies used, such as the FDR of the activation map or the histogram analysis.

The study of the effects of combining datasets from different sources is especially interesting because it shows that, if it is not strictly controlled, important biases can be induced in the final dataset. A typical solution for the lack of samples of a given class is to compile different datasets into one that collects all the categories to study, as the recent COVID-19 datasets. In particular, the widely used COVIDcxr dataset, built from different sources, might in fact have included significant biases that inadvertently affected the results published. This kind of heterogeneous dataset often mix observations coming from very diverse equipment, acquisition protocols and processing software. In that context, features found by Deep Convolutional Networks in the images, including the background areas, are enough to get a good classification rate, whilst the actual performance of the classifier for the clinical task attempted can be much lower.
